# Development and Application of a Nanobody-Based Competitive ELISA for Detecting Antibodies against Hepatitis E Virus from Humans and Domestic Animals

**DOI:** 10.1128/spectrum.03607-22

**Published:** 2023-06-22

**Authors:** Yiyang Chen, Meimei Zhang, Tianxiang Chen, Jiaxi Wang, Qin Zhao, En-Min Zhou, Baoyuan Liu

**Affiliations:** a Department of Preventive Veterinary Medicine, College of Veterinary Medicine, Northwest A&F University, Yangling, Shaanxi, China; b Scientific Observing and Experimental Station of Veterinary Pharmacology and Diagnostic Technology, Ministry of Agriculture, Yangling, Shaanxi, China; Quest Diagnostics

**Keywords:** hepatitis E virus (HEV), cELISA, antibodies, humans, domestic animals

## Abstract

Hepatitis E virus (HEV) is a zoonotic pathogen that is widespread worldwide. At present, most enzyme-linked immunosorbent assay (ELISA) kits only detect antibodies against human HEV. In this study, a nanobody-horseradish peroxidase (HRP) fusion protein-based competitive ELISA (cELISA) with more convenience and spectral characteristics for HEV antibody detection was developed and used to detect HEV IgG in various species. First, 6 anti-swine HEV capsid protein nanobodies were screened using phage display technology from an immunized Bactrian camel. Then, HEV-Nb67-HRP fusions were expressed and used as a probe for developing a cELISA. The cutoff value of the cELISA was 17.8%, and there was no cross-reaction with other anti-swine virus antibodies, suggesting that the cELISA had good specificity. The intra-assay and interassay coefficients of variation (CVs) were 1.33 to 5.06% and 1.52 to 6.84%, respectively. The cELISA and Western blot showed a higher coincidence rate (97.14%, kappa value = 0.927) than cELISA and indirect ELISA (95.00%, kappa value = 0.876) in clinical swine serum samples. Finally, the seroprevalence of HEV IgG in humans, pigs, rabbits, cows, and goats was 30.67%, 19.26%, 8.75%, 27.59%, and 18.08%, respectively, suggesting that cELISA may have a broader scale for mammalian HEV antibody detection. These results suggest that the newly developed cELISA was rapid, low-cost, reliable, and useful for the serological evaluation of current HEV.

**IMPORTANCE** HEV is thought to be a zoonotic infection and is widespread worldwide; it is beneficial to establish a more convenient and spectral method for HEV antibody detection. In this study, a convenient, time-saving, reproducible, highly sensitive, specific, and novel nanobody-based cELISA was developed and can be used to detect IgG antibodies against mammalian HEV. It provides a new technique for serological evaluation and ELISA-based diagnosis of HEV infection.

## INTRODUCTION

Hepatitis E is an important public health concern in many developing countries and occurs sporadically in some developed countries (1). Although the overall mortality rate associated with the disease is low, it is reportedly as high as 30% for infected pregnant women (2). The causative agent of hepatitis E, hepatitis E virus (HEV), is a single-stranded, positive-sense RNA virus that circulates in the blood as quasi-enveloped particles or shedding in the feces as nonenveloped particles (3). The virus is transmitted primarily by the fecal-oral route in areas with poor sanitary conditions. However, there have been cases of HEV infection by consuming HEV-infected meat products in developed countries (4). To date, increasing evidence has demonstrated that the transmission of HEV is zoonotic (5). Various species of domestic and wild animals have been found with a high prevalence of anti-HEV antibodies, and pigs have been considered reservoirs of HEV (6–11).

HEV belongs to the family *Hepeviridae*, which contains two genera, *Orthohepevirus* (species A to D) and *Piscihepevirus*. *Orthohepevirus* A is classified into eight genotypes (1 to 8) (12). HEV-1 and HEV-2 are considered responsible for human hepatitis E epidemics worldwide. HEV-3 and HEV-4 usually induce sporadic infection and are zoonotic, while HEV-5 and HEV-6 were discovered in wild boar, and HEV-7 and HEV-8 were isolated from camels (13). Although HEV shows high genetic diversity, only one serotype has been identified thus far (14). The 7.2-kb HEV genome contains three main open reading frames (ORFs), ORF1, ORF2, and ORF3 (15), and a novel ORF4 has been found only in the genotype 1 HEV genome (16). ORF2 encodes a capsid protein that contains the primary antigenic epitopes and is regarded as the antigen for serological diagnosis of HEV infection (17). At present, there is a widely recognized commercial indirect enzyme-linked immunosorbent assay (ELISA) kit that only detects antibodies against human HEV (18). Generally, secondary antibodies needed to be replaced, or the laboratory needs to establish its own method for other species detection (19). Therefore, it is necessary to develop a more convenient and spectral method for HEV antibody detection.

Recently, heavy chain-only antibodies (HcAbs), nanobodies (15 kDa), have been shown to be highly stable, easy to produce, and inclined to associate with concave-shaped epitopes (20). Therefore, compared with conventional antibodies, it is becoming a more hopeful tool for the diagnosis and therapy of various diseases.

In the present study, nanobodies against the truncated swine HEV ORF2 protein were screened by phage display technology from a Bactrian camel immunized with the recombinant truncated ORF2 protein. With the platform (21), a nanobody-horseradish peroxidase (HRP) fusion protein-based competitive ELISA for convenient and faster detection of anti-HEV antibodies was developed. Furthermore, anti-HEV antibodies were detected in the sera of humans, pigs, rabbits, cows, and goats with competitive ELISA (cELISA), suggesting that this method has a broader scale for mammalian HEV antibody detection.

## RESULTS

### Screening and sequencing of nanobodies against the S268 protein.

**FIG 1:**
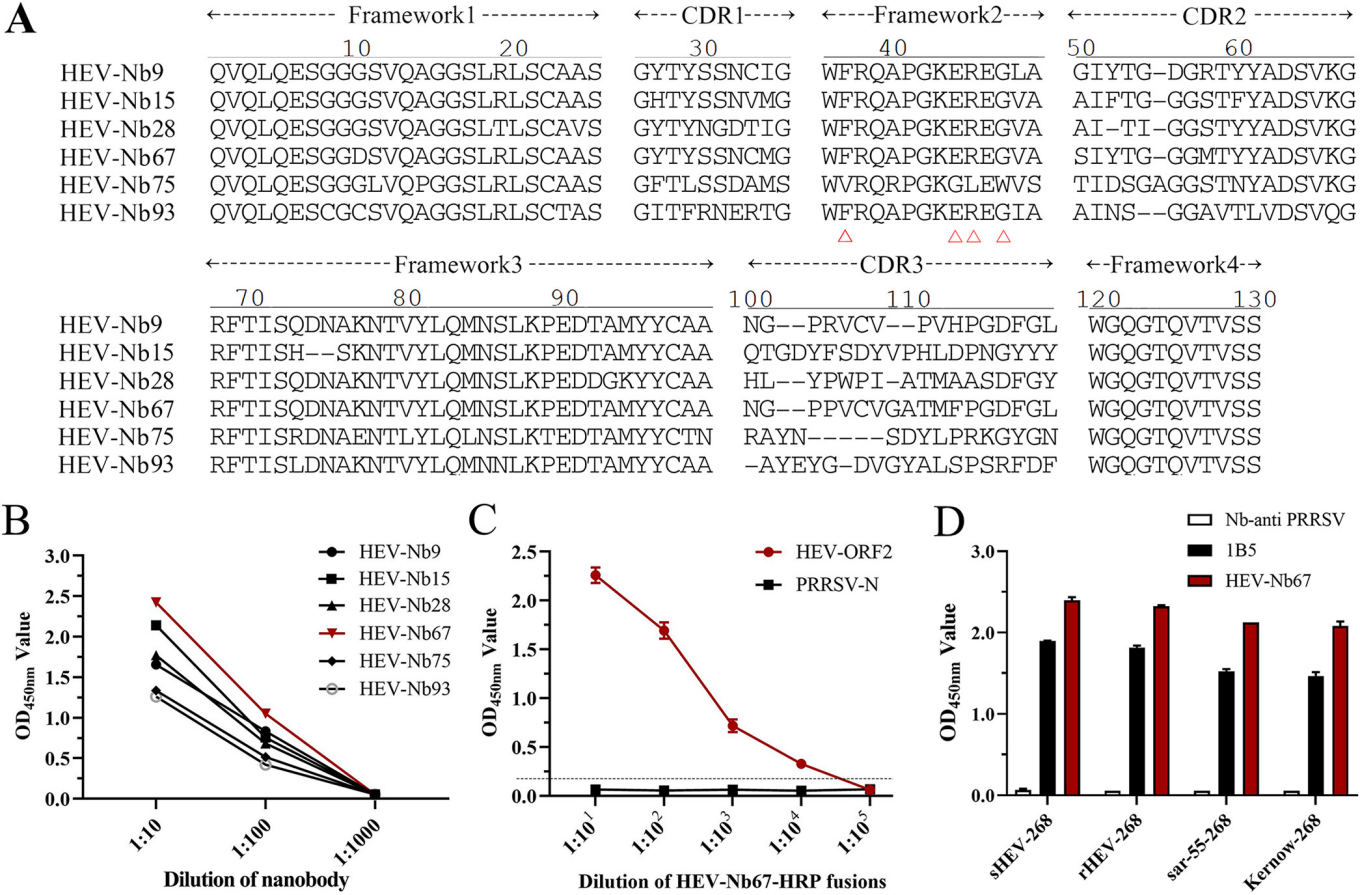
Screening and eukaryotic of specific nanobody against the S268 protein. (A) Alignment of the amino acid sequences of 6 screened nanobodies. The conserved residues (hydrophilic amino acids) at positions 37, 44, 45, and 47 are indicated by red triangles. (B) Titration of the 6 screened nanobodies binding with the S268 protein. (C) Detection of CHD-SD-sHEV-Nb67-HRP fusion reaction with S268 protein using direct ELISA. The dotted line represents three times the negative OD_450_ value. (D) The fusions interact with S268, rHEV-268, Sar-55-268, and Kernow C1-268 protein by direct ELISA. The 1B5 (a monoclonal antibody against HEV ORF2) and Nb-anti PRRSV were used as positive and negative controls, respectively.

On the basis of previous methods, a phage display VHH library was constructed from an S268 protein-immunized camel, and the phage particles were strongly enriched after 3 rounds of panning (Table 1). Subsequently, the periplasmic extracts from 96 individual colonies were expressed and screened for binding to the S268 protein using indirect ELISA (iELISA). Amino acid sequence analysis revealed that 6 nanobodies (Nb9, Nb15, Nb28, Nb67, Nb75, and Nb93) were identified based on the CDR3 region (Fig. 1A). Since Nb67 showed the strongest reaction with the S268 protein (Fig. 1B), it was chosen and expressed for further development of a cELISA using nanobody-HRP fusions as reagents.

**TABLE 1:** Enrichment of nanobodies against the S268 protein from the phages during three rounds of panning^*a*^

Round of panning	Phage input (PFU/well)	Phage output/P (PFU/well)	PBS output/N (PFU/well)	Recovery rate (P/input)	Enrichment (P/N)
1	5 × 10^10^	3.6 × 10^4^	3.45 × 10^2^	7.2 × 10^−7^	1.04 × 10^2^
2	5 × 10^10^	2.44 × 10^7^	1.16 × 10^5^	4.88 × 10^−5^	2.1 × 10^2^
3	5 × 10^10^	3.39 × 10^7^	7.1 × 10^4^	9 × 10^−4^	1.33 × 10^3^

a PBS, phosphate-buffered saline.

### Expression of CHD-SD-sHEV-Nb67-HRP fusion protein in HEK293T cells.

After the CHD-SD-sHEV-Nb67 VHH gene was inserted into the pCMV-N1-HRP vector, the CHD-SD-sHEV-Nb67-HRP fusion protein was successfully expressed in HEK293T cells with a titer that exceeded 1:10^4^ (three times for a negative optical density at 450 nm [OD_450_] value was considered positive) in the medium (Fig. 1C). In addition, the ELISA showed that the fusions also reacted with capsid proteins (amino acids [aa] 393 to 660) of the genotype 3 rabbit HEV strain (CHN-SX-rHEV) and genotype 1 and 3 human HEV strains (Sar-55 and Kernow C1) (Fig. 1D).

### Development of competitive ELISA.

The checkerboard titration assay showed that the optimal coating concentration of S268 protein was 1 μg/mL, and the dilution of HEV-Nb67-HRP fusions was 1:2^8^ (Table 2). The best dilution of sera was 1:10 (Table 3). Subsequently, a checkerboard assay showed that the optimal incubation time of the mixture with S268 protein was 45 min, and the colorimetric reaction time was 15 min (Table 4). The difference value of positive-to-negative (P/N value between 1:5 and 1:10 was less than 0.01); thus, the higher dilution ratio of 1:10 was preferred under the condition that the difference of P/N values was not obvious. Similarly, the shorter reaction time (45 min) was chosen in this cELISA.

**TABLE 2:** Determination of the optimal coating amount of S268 protein and the optimal dilution of HEV-Nb67-HRP fusions using direct ELISA

Amt of HEV-ORF2C protein (μg/mL)	Dilution of HEV-Nb67-HRP fusions in medium							
	1:2^4^	1:2^5^	1:2^6^	1:2^7^	1:2^8^	1:2^9^	1:2^10^	1:2^11^
4	2.664	2.601	2.499	2.203	2.042	1.502	1.028	0.541
2	2.421	2.372	2.2	1.966	1.439	0.844	0.493	0.261
1	2.224	1.874	1.77	1.448	**0.989** ^*a*^	0.601	0.3	0.179
0.5	1.958	1.446	1.119	0.855	0.484	0.227	0.14	0.084

a The optimal amount of S268 protein and dilution of HEV-Nb67-HRP were selected when the OD_450_ value of the direct ELISA was approximately 1.0.

**TABLE 3:** Determination of the optimal dilution of tested pig sera for cELISA

Serum no.	Serum type	1:80	1:40	1:20	1:10	1:5
1	Positive	0.773	0.501	0.476	0.445	0.413
	Negative	1.022	0.992	1.073	1.046	0.977
	P/N	0.756	0.505	0.444	**0.425** ^*a*^	0.423
2	Positive	0.505	0.374	0.217	0.181	0.167
	Negative	1.057	1.07	1.058	1.006	0.945
	P/N	0.478	0.35	0.205	**0.180**	0.177
3	Positive	0.334	0.276	0.217	0.156	0.142
	Negative	1.100	1.032	0.973	1.065	1.013
	P/N	0.304	0.267	0.223	**0.146**	0.14

a Three positive and negative swine sera were separately used for cELISA detection. The dilutions of sera were 1:5, 1:10, 1:20, 1:40, and 1:80. The best dilution was selected when the OD_450_ value of positive-to-negative (P/N) sera was smallest.

**TABLE 4:** Optimized incubation time of the mixture containing swine sera and HEV-Nb67-HRP fusions and optimal time for the colorimetric reaction after adding TMB using a checkerboard assay with cELISA

Time (min) of color reaction	Serum type	Incubation time (min) of swine sera and HEV-Nb67-HRP fusions			
		15	30	45	60
10	Positive	0.078	0.098	0.122	0.163
	Negative	0.226	0.432	0.552	0.815
	P/N	0.343	0.226	0.221	0.200
15	Positive	0.093	0.125	0.186	0.193
	Negative	0.339	0.578	0.989	1.048
	P/N	0.274	0.216	**0.188** ^*a*^	0.184

a The Optimized incubation time of the mixture containing swine sera and HEV-Nb67-HRP fusions and optimal time for the colorimetric reaction were selected when the OD_450_ value of positive-to-negative (P/N) sera was smallest.

After optimization, the cELISA was carried out as follows. First, the plates were coated with S268 protein (1 μg/mL, 100 μL/well) in phosphate buffer (PB) (pH 7.5) and incubated at 4°C. The next day, the plates were blocked with 200 μL blocking buffer at room temperature (RT) for 1 h after washing three times. Then, the washing operation was performed again, and the wells were added to 100-μL testing mixtures containing 10-μL testing pig serum samples and 90 μL HEV-Nb49-HRP fusions and incubated at RT for 45 min. After washing, 100 μL tetramethylbenzidine (TMB) was added, and the plates were incubated in the dark for 15 min at RT. As a last step, 3 M H_2_SO_4_ (50 μL/well) was added to stop the reaction, and the OD_450_ value was read by an automated ELISA plate reader.

### Cutoff values for the competitive ELISA.

The 188 negative pig sera for anti-swine HEV antibodies were assayed by cELISA to determine the cutoff values. The results showed that the average percent competitive inhibition (PI) value of these serum samples was 3.7%, with a standard deviation (SD) of 4.7%. Thus, the cutoff value was 17.8% (3.7% + 3 × 4.7%) for the developed cELISA. If the PI value of the tested pig serum was equal to or greater than 17.8%, it was considered positive.

### Sensitivity, specificity, and reproducibility of the competitive ELISA.

**FIG 2:**
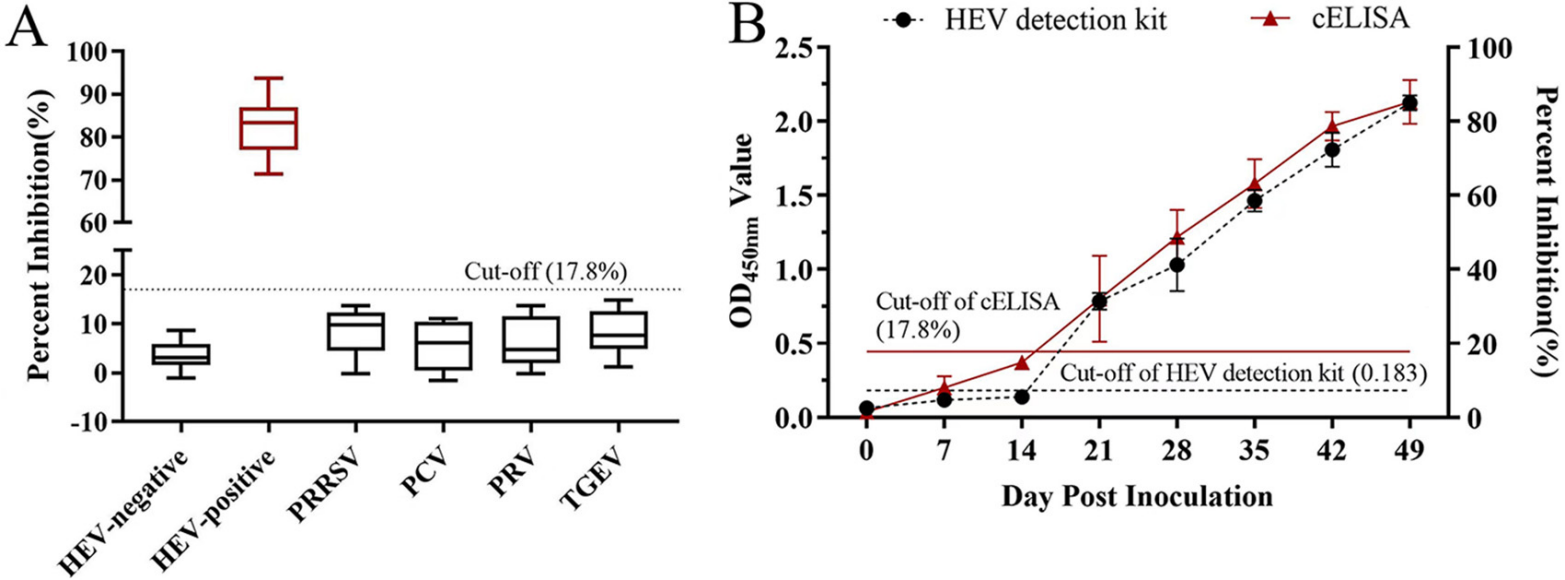
Sensitivity and specificity of the developed cELISA using CHD-SD-sHEV-Nb67-HRP as a probe. (A) Detection of antibodies against swine HEV in serial sera from pigs challenged with CHD-SD-sHEV by cELISA and a commercial ELISA kit. (B) Evaluation of the cELISA detecting antibodies against other swine disease viruses, including PRRSV, PCV, PRV, and TGEV.

To determine the specificity of cELISA, the positive serum against other swine viruses was negative for detection (PI values from 0.23% to 14.93%) (Fig. 2A). A total of 24 sequential sera were assayed to determine the sensitivity of the developed cELISA. The results showed that 3 healthy pigs (HEV antibody and RNA both negative) seroconverted at 21 days postinfection (dpi) by using both cELISA and commercial ELISA kits (Wantai Biological Pharmacy Co., Beijing, China) for detection, and all pigs were still positive for anti-swine HEV antibodies until 49 dpi (Fig. 2B). In addition, the reproducibility results showed that the intra-assay coefficient of variation (CV) of the PI was 1.33 to 5.06% with a median value of 3.81%, and the interassay CV was 1.52 to 6.84% with a median value of 4.93%.

### Agreements of cELISA with a commercial ELISA kit and with Western blot analysis.

To determine if the developed cELISA can be used to test clinical samples, 280 clinical swine serum samples collected from healthy pigs of different ages in five flocks were assayed by the developed cELISA, commercial ELISA kit, and Western blotting. The results showed that the cELISA and commercial ELISA kit with Western blot analysis coincided in 266/272 of the 280 serum samples with an agreement rate of 95.00% and 97.14%, respectively (Table 5). Moreover, the statistical analysis showed that the agreement of cELISA and Western blot (kappa = 0.927) was higher than that between cELISA and the commercial ELISA kit (kappa = 0.876) (Table 5), and there were no significant differences (all kappa values were >0.4).

**TABLE 5:** Comparisons of the developed cELISA with commercial ELISA kit and Western blotting by testing clinical swine serum samples^*a*^

Serum no.	cELISA	Commercial ELISA kit		Agreement (%)	Kappa value	Western blotting		Agreement (%)	Kappa value
		No. positive	No. negative			No. positive	No. negative		
71	No. positive	71 (A)	0 (B)	95.00	0.876	71 (A)	0 (B)	97.14	0.927
209	No. negative	14 (C)	195 (D)			8 (C)	201 (D)		

a Percent agreement = (A+D)/280 × 100.

### Detection of HEV IgG in humans and other species.

**FIG 3:**
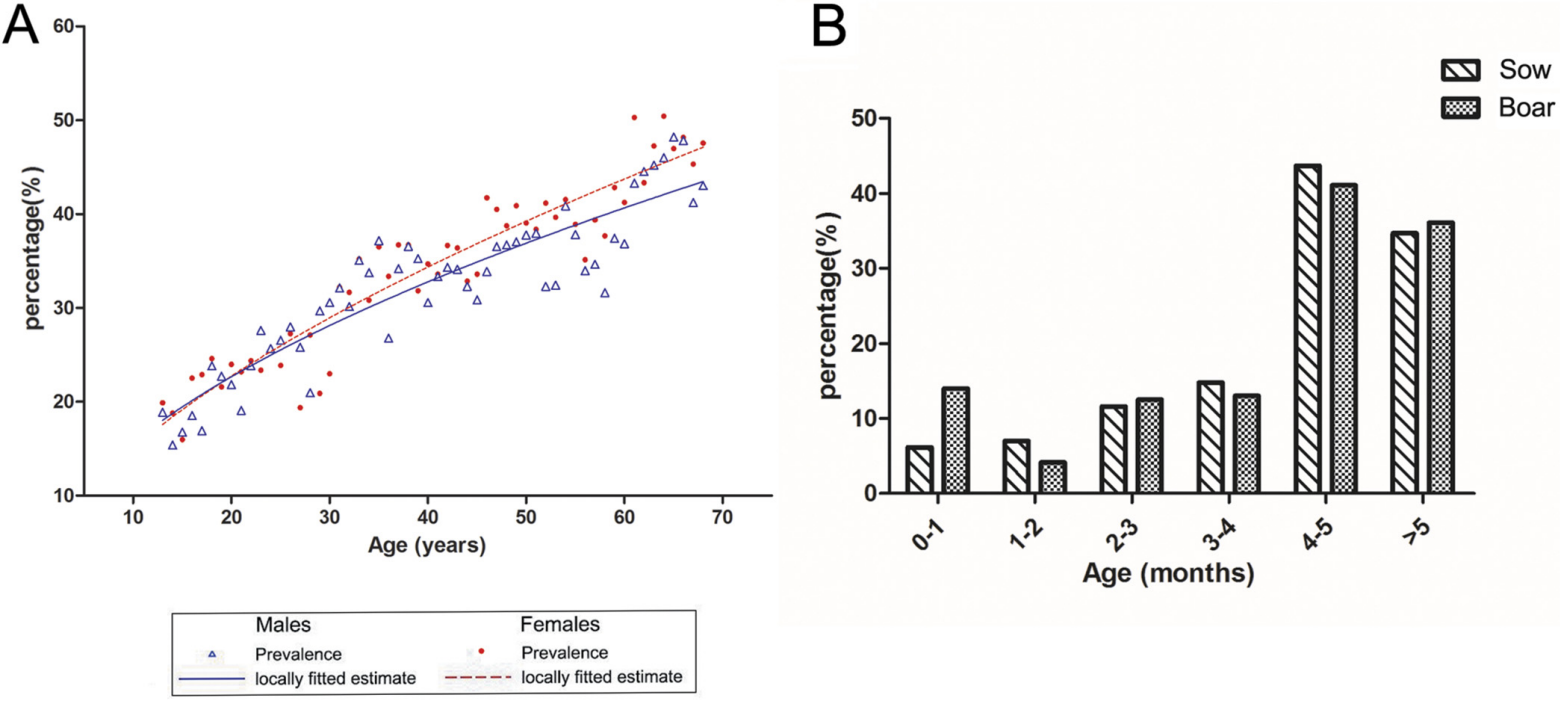
Estimated anti-HEV IgG seroprevalence in humans (A) and pigs (B) by age and sex.

A total of 2,344 humans (age range, 13 to 87 years) were enrolled in the study. The results showed that the overall seroprevalence in humans was 30.67%, and the positive rates of females and males were 30.51% and 30.81%, respectively (Table 6). No significant difference in HEV seroprevalence was found between the sexes (*P* > 0.05). Analysis of age was more complicated by the fact that studies evaluating whether anti-HEV IgG seropositivity increases with age. Based on the age of these participants, they were divided into six age groups, 13 to 17 years (*n* = 410), 18 to 22 years (*n* = 395), 22 to 30 years (*n* = 399), 30 to 45 years (*n* = 395), 45 to 60 years (*n* = 374), and >60 years (*n* = 371). The positivity rate increased from 19.27% (13- to 17-year age group) to 46.36% (>60 age group) (odds ratio, 3.621; 95% confidence interval [CI], 2.632 to 4.983). The observed seroprevalence in females and males increased at rates of 0.541% and 0.465%, respectively, with each year of age (Fig. 3A).

**TABLE 6:** Anti-HEV in human and other animals in Shaanxi province according to different age groups, genders, and areas

Characteristic	Sample size	No. positive	Positivity rate (95% CI)	OR (95% CI)^*a*^	χ^2^	*P* value
Humans						
Gender						
Male	1,098	335	30.51 (0.2777–0.3325)	Ref		
Female	1,246	384	30.81 (0.2825–0.3337)	0.986 (0.827–1.175)	0.026	0.893
Age						
13–17	410	79	19.27 (15.45–23.09)	ref		
18–22	395	92	23.29 (19.12–27.46)	1.272 (0.907–1.785)	1.946	0.169
22–30	399	100	25.06 (20.81–29.31)	1.401 (1.003–1.957)	3.94	0.051
30–45	395	135	34.17 (29.49–38.85)	2.176 (1.577–3.001)	22.91	<0.01^*b*^
45–60	374	141	37.7 (32.79–42.61)	2.536 (1.837–3.500)	32.92	<0.01^*b*^
>60	371	172	46.36 (41.29–51.43)	3.621 (2.632–4.983)	65.55	<0.01^*b*^
Total	2344	719	30.67 (28.80–32.54)			
Swine						
Gender						
Sow	448	86	19.19 (15.54–22.84)	Ref		
Boar	232	45	19.39 (14.30–24.48)	1.013 (0.678–1.513)	0.004	0.513
Age (mo)						
0–1	210	18	8.57 (4.78–12.35)	Ref		
1–2	103	6	5.83 (1.31–10.35)	0.66 (0.254–1.716)	0.736	0.5
2–3	67	8	11.94 (4.18–19.70)	1.446 (0.598–3.495)	0.678	0.47
3–4	77	11	14.29 (6.47–22.11)	1.778 (0.798–3.959)	2.025	0.184
4–5	118	51	43.22 (34.28–52.16)	8.119 (4.434–14.869)	54.602	<0.01^*b*^
>5	105	37	35.24 (26.10–44.38)	5.804 (3.099–10.870)	34.54	<0.01^*b*^
Total	680	131	19.26 (16.30–22.22)			
Rabbit	80	7	8.75 (6.56–14.94)	NA		
Cow	58	16	27.59 (16.08–39.10)	NA		
Goat	177	32	18.08 (12.41–23.75)	NA		

a OR, odds ratio; CI, confidence interval; χ^2^, chi-square test; ref, reference; NA, not analyzed.

b Statistically significant.

A total of 680 serum samples from 11 flocks were used in this study for statistical analysis. The total positive rate of domestic pigs was 19.26%, as shown in Table 6. The chi-square analysis showed that there was no significant difference between sows and boars (*P* > 0.05). However, significant differences were observed in different age groups. As shown in Table 6 and Fig. 3B, seroprevalence ranged from 5.83% to 43.22% in the various age groups, with the highest seroprevalence observed in the 4- to 5-month group, followed by the >5-month age group (35.24%, 95% CI).

Eighty rabbit, 58 cow, and 177 goat serum samples were also used to investigate antibodies against HEV, and the positive rates were 8.75%, 27.59%, and 18.08%, respectively (Table 6).

## DISCUSSION

HEV is considered zoonotic in a broad range of animals and poses a risk to the growing population of immunocompromised people (22). However, there is a widely recognized commercial kit to detect antibodies against HEV only for humans (18). Currently, secondary antibodies need to be replaced, or the laboratory has established its own method for other species detection (19, 23). Therefore, it is advantageous to develop a more convenient and spectral method for HEV antibody detection with nanobody. Meanwhile, anti-HEV IgG has been shown among pigs and other animals in several countries of HEV endemicity and nonendemicity, including China (24, 25). Since hepatitis E broke out in Xinjiang from July 1986 to April 1988, this disease has become a widespread epidemic in China (26). In this study, a nanobody-HRP fusion was used to develop cELISA for detecting antibodies against HEV for the first time, and no secondary antibody is required for detection in this method, which greatly saves costs and time. Meanwhile, the sera of humans and different species, including swine, rabbit, goat, and cow, were used in this cELISA for anti-HEV IgG detection.

In our previous study, a blocking ELISA for HEV detection had been developed with HRP-labeled monoclonal antibody (MAb) as probe (23); however, HRP-unlabeled MAb cannot be removed completely, and the inefficient labeling may lead to false positives. The nanobody-HRP fusion protein-based competitive ELISA avoids the above problem and is more convenient for faster detection of anti-HEV antibodies.

HEV ORF2 encodes the virus capsid protein and contains the main immunodominant epitopes of virions (27). HEV has a single serotype and four major genotypes (HEV-1 to HEV-4), with >90% amino acid sequence identity among them (28). In this research, the direct ELISA results showed that Nb67 bound to the ORF2 protein of swine HEV-4, human HEV-1 (Sar-55), human HEV-3 (Kernow C1), and rabbit HEV-3 (Fig. 1D). These results reveal that the same binding ability of Nb67 may apply to other HEV genotype strains. Meanwhile, the seroprevalence of HEV IgG in humans, pigs, rabbits, cows, and goats was 30.67%, 19.26%, 8.75%, 27.59%, and 18.08%, respectively, with cELISA detection, which further indicated that cELISA had a broader scale for mammalian HEV antibody detection.

Our results showed that HEV seroprevalence in humans increased with age, which has been confirmed by previous reports (29–31). In this study, individuals aged >60 years were 2 to 3 times more likely to be HEV positive than those aged <30 years, and one possible reason may be their accumulated lifetime exposure to the virus. Previous studies showed that the seropositivity rate of HEV was significantly higher in males than females (32), while some showed higher rates in females (33), and other studies showed no significant difference between male and female populations (29, 34), which was in accordance with this study. This discrepancy may be caused by the different populations and HEV genotypes.

Pigs have been confirmed to be major reservoirs of HEV-3 and HEV-4. In China, many serological investigations of the status of HEV infection in domestic pigs have been carried out. Previous studies documented that the seroprevalence of anti-HEV IgG antibodies was 66.4% in Shandong (35), 68.3% in Hunan (36), and 63.9% in Shanghai and Jiangsu (37), and there was an average seroprevalence of 78.8% in Beijing, Henan, Zhejiang, Guangdong, and Hubei (25). Some studies showed that the seroprevalence of pig sera collected from 26 provinces across the country was up to 82.2% (38). These investigations suggested that HEV infection is endemic in domestic pig populations in some regions of China. The discrepancy in the antibody prevalence rates can be attributed to the different assay systems and to the different calculation methods used to decide a cutoff value. In this study, the seroprevalence of swine in northwestern China was 19.26% (95% CI, 16.30% to 22.22%), which was lower than the countrywide level and that in eastern China. The reason may lie in the fact that the pork industry in northwestern China began more recently, is still of small scale, and incorporates the highest biosafety practices. Therefore, it is advantageous for the pork industry to encourage higher biosafety efforts to prevent HEV virus infection. In addition, the proportion of anti-HEV IgG-positive pigs was obviously higher among pigs 4 to 5 months of age, which was in line with previous reports (39, 40) and consistent with the natural infection of pigs aged approximately 10 to 12 weeks, as maternal antibodies to HEV might persist up to 9 weeks and confer resistance to viral infection in young pigs (1).

In summary, a nanobody Nb67-based cELISA has been developed and used to detect IgG antibodies against mammalian HEV. This method is convenient, time-saving, reproducible, spectral, highly sensitive, and specific. The HEV seroprevalence results showed that humans and domestic species were still at risk of HEV infection, suggesting that necessary measures must be taken to prevent and control HEV infection in humans, swine, and other species. Simultaneously, further investigations are being conducted to identify the HEV genome in swine and other species with ongoing HEV infections as well as in sewage samples of animal origin for the genetic characterization of the virus.

## MATERIALS AND METHODS

### Screening and eukaryotic activity of specific nanobodies against the S268 protein.

A 4-year-old male Bactrian camel was immunized 5 times (2-week intervals) with 2 mg S268 protein (the truncated ORF2 protein of CHD-SD-sHEV, aa 393 to 660) by subcutaneous injection as described in previous studies (41). The Freund’s complete (first immunization) and incomplete adjuvant (the next four immunizations) with equal volumes as S268 protein was used. Peripheral blood mononuclear cells (PBMCs) were isolated 4 days after the last immunization. Total RNA was extracted (Qiagen Bioinformatics, Germany) and reverse transcribed into cDNA. Subsequently, the 400-bp band-recovered VHH fragments were amplified using nested PCR (21) and inserted into the modified pCANTAB 5E vector. Eventually, the recombinant plasmid was electrotransferred into Escherichia coli TG1 competent cells, and the cells were spread on Luria-Bertani (LB) agar plates containing 2% glucose and 100 μg/mL ampicillin and cultured at 37°C for 8 to 12 h. Colonies were scraped and tested with primers as previously described (42) and then stored at −80°C in LB supplemented with 20% glycerol.

To screen specific nanobodies against the S268 protein, phage rescue and titration were performed as previously described (21). Briefly, the plates were coated with S268 protein (5 μg/well). After three rounds of biopanning, the enrichment of specific phage particles was evaluated with polyclonal phage ELISA. The 96 single colonies were randomly selected for further analysis. Expression of nanobodies was induced by 1 mmol/L IPTG (isopropyl-β-d-thiogalactopyranoside; TaKaRa, Japan). The nanobodies were extracted using an osmotic shock protocol from the periplasm (43), and their capacity to bind with the S268 protein was tested by indirect ELISA (iELISA). Subsequently, all VHH genes from the positive clones were sequenced and classified according to their CDR3 sequence. Finally, the best binding ability of the nanobody was selected as a candidate reagent for establishing the following cELISA.

The candidate nanobody gene (Nb67; GenBank accession number OQ679741) was inserted into the pCMV-N1-HRP vector (the expression platform of nanobody-HRP fusions), and then the recombinant plasmid was transfected into HEK293T cells. After 48 h, the cell culture medium was collected and filtered through 0.45-μm pore cellulose acetate membranes for direct use.

### Development of the competitive ELISA.

First, different concentrations of S268 proteins were coated (0.5 μg/mL, 1 μg/mL, 2 μg/mL, and 4 μg/mL), and the dilution ratios of nanobody-HRP fusions were from 1:2^4^ to 1:2^11^. The final optimal conditions were determined to produce approximate OD_450_ values of 1.0 with direct ELISA by checkerboard titration (21). Subsequently, three separate anti-swine HEV antibody-negative and -positive pig sera were diluted 1:5, 1:10, 1:20, 1:40, and 1:80 for cELISA testing. The optimal serum dilution was determined when the smallest ratio of OD_450_ values between the positive and negative serum (P/N) was obtained. Finally, the incubation time of the mixtures was set to 15, 30, 45, and 60 min, and after tetramethylbenzidine (TMB) was added, the colorimetric reaction time was set to 10 and 15 min. The two optimal reaction conditions were chosen as those when the smallest ratio of P/N was obtained.

After optimizing the above-described conditions, the 96-well ELISA plate was coated with the optimum concentration of S268 protein in the PB (phosphate buffer at pH 7.5, consisting of NaH_2_PO_4_ and Na_2_HPO_4_) and then incubated at 4°C. The next day, the plate was blocked with blocking buffer for 1 h at room temperature (RT) after washing three times. Then, the washing operation was performed again, and 100 μL of the test mixtures was added to each well (the optimal dilutions of serum sample and nanobody-HRP fusions) and then incubated for the optimal time at RT. After washing again, 100 μL TMB was added and incubated at RT for the optimal time. As the last step, 3 M H_2_SO_4_ (50 μL/well) was added to stop the reaction, and the OD_450_ value was read by an automatic ELISA microplate reader.

### Validation of the competitive ELISA.

The percent competitive inhibition (PI) was calculated using the following formula: PI (%) = [1 − (OD_450_ value of testing/negative serum sample)] × 100%. The cutoff value was determined by the mean PI of 188 negative serum samples plus 3 standard deviations (SDs) with 99% confidence.

To evaluate whether the development of cELISA had cross-reactivity with other positive sera against the swine virus, including porcine reproductive and respiratory syndrome virus (PRRSV), porcine circovirus (PCV), porcine pseudorabies virus (PRV), and transmissible gastroenteritis virus (TGEV), 52 serum samples were tested. To further evaluate the sensitivity of cELISA, a panel of 24 sequential swine serum samples from 3 challenged pigs was tested by cELISA and a commercial ELISA kit (changed HRP-conjugated goat anti-swine IgG as secondary antibodies).

To evaluate the repeatability of the cELISA, three separate negative and positive serum samples were selected. Each sample was added to three replicates in a plate and used to determine the intra-assay coefficient of variation (CV), and three different plates on different occasions were used to calculate the interassay CV.

### Comparisons of cELISA with a commercial ELISA kit and Western blotting.

A total of 280 clinical serum samples were collected from healthy pigs of different ages in five flocks of Shaanxi Province and tested by the developed cELISA, commercial ELISA kit, and Western blotting.

### Serum samples.

To determine the cutoff value of the cELISA, a total of 188 negative pig sera for anti-swine HEV antibodies (detected by a commercial ELISA kit and the laboratory-established ELISA) were used in this study. A panel of 24 sequential pig serum samples (collected from 3 challenged pigs 0, 7, 14, 21, 28, 35, 42, and 49 days postinoculation) was used to validate the sensitivity of cELISA. To evaluate whether the developed cELISA test had cross-reactivity with other swine virus antisera, 52 serum samples raised were investigated against other swine viruses, including PRRSV (*n* = 20), PCV (*n* = 10), PRV (*n* = 12), and TGEV (*n* = 10). A total of 280 clinical swine serum samples collected from healthy pigs of different ages in five flocks were used to evaluate the consistency of cELISA with other testing methods.

In addition, serum from various species was used for HEV antibody detection to verify that cELISA has a wide range of applications. A total of 2,344 human serum samples were collected from apparently healthy individuals, including 410 aged 13 to 17 years, 395 aged 18 to 22 years, 399 aged 22 to 30 years, 395 aged 30 to 45 years, 374 aged 45 to 60 years, and 371 aged above 60 years. The numbers of serum samples from females and males of different ages are shown in Table 6. Serum samples from 680 pigs (11 herds of industrialized farms), 80 rabbits, 58 cattle, and 177 goats were also used for anti-HEV antibody detection.

### Statistical analysis.

The kappa values were calculated to estimate the coincidence between cELISA and commercial ELISA kits and between cELISA and Western blotting, and the prevalence of antibodies in different age groups for human and pig serum samples and in different animal species was compared with the chi-square test using statistical software SPSS version 20. Statistical significance was defined at *P* values of <0.05.

### Ethical considerations.

All owners of the animals referred to in this study gave permission for their animals’ sera to be used in this study. The study objectives and protocols of human sera were explained to the local hospitals from Shaanxi Province in China. Written consent was obtained prior to sample collection. The study protocol was approved by the Northwest Agricultural and Forestry University Experimental Animal Welfare Ethics Committee.
